# Real‐world complication burden and disease management paradigms in transfusion‐related β‐thalassaemia in Greece: Results from ULYSSES, an epidemiological, multicentre, retrospective cross‐sectional study

**DOI:** 10.1002/jha2.695

**Published:** 2023-05-23

**Authors:** Antonis Kattamis, Ersi Voskaridou, Sophia Delicou, Evangelos Klironomos, Ioannis Lafiatis, Foteini Petropoulou, Michael D. Diamantidis, Stylianos Lafioniatis, Loukia Evliati, Eleni Kapsali, Kiki Karvounis‐Marolachakis, Despoina Timotheatou, Chrysoula Deligianni, Panagiotis Viktoratos, Alexandra Kourakli

**Affiliations:** ^1^ First Department of Pediatrics Thalassemia Unit National and Kapodistrian University of Athens Athens Greece; ^2^ Expertise Center in Rare Haematological Diseases‐Haemoglobinopathies General Hospital of Athens “Laikon” Athens Greece; ^3^ Thalassemia and Sickle Cell Unit General Hospital of Athens “Hippocrateion” Athens Greece; ^4^ Thalassemia and Sickle Cell Unit General Hospital of Heraklion “Venizelion” Heraklion Greece; ^5^ Thalassemia and Sickle Cell Unit General Hospital of Mytilene “Vostanio” Mytilene Greece; ^6^ Thalassemia Unit General Hospital of Athens “Georgios Gennimatas” Athens Greece; ^7^ Thalassemia and Sickle Cell Disease Unit General Hospital of Larissa “Koutlimbaneio & Triantafylleio” Larissa Greece; ^8^ Thalassemia and Sickle Cell Unit General Hospital of Volos “Achilopouleio,” Volos Greece; ^9^ Thalassemia and Sickle Cell Unit General Hospital of Athens “Evaggelismos” Athens Greece; ^10^ Department of Hematology University Hospital of Ioannina Ioannina Greece; ^11^ Medical Department Genesis Pharma SA Athens Greece; ^12^ Medical Department Bristol‐Myers Squibb Company SA Athens Greece; ^13^ Department of Internal Medicine Hematology Division University General Hospital of Patras Patras Greece

**Keywords:** complications, epidemiological, real‐world, transfusion‐related β‐thalassaemia

## Abstract

Patients with transfusion‐dependent beta (β)‐thalassaemia experience a broad range of complications. ULYSSES, an epidemiological, multicentre, retrospective cross‐sectional study, aimed to assess the prevalence and severity of treatment and disease complications, capture disease management and identify predictors of complications in patients with transfusion‐dependent β‐thalassaemia, treated in routine settings in Greece. Eligible patients were adults diagnosed with β‐thalassaemia ≥12 months before enrolment and having received ≥6 red blood cell (RBC) units (excluding elective surgery) with no transfusion‐free period ≥35 days in the 24 weeks before enrolment. Primary data were collected at a single visit and through chart review. Between Oct 21, 2019, and Jun 15, 2020, 201 eligible patients [median (interquartile range, IQR) age 45.7 (40.2–50.5) years; 75.6% > 40 years old; 64.2% female] were enrolled, a mean (standard deviation) of 42.9 (7.8) years after diagnosis. Median (IQR) age at diagnosis and RBC transfusion initiation were 0.8 (0.4–2.8) and 1.3 (1.0–5.0) years, respectively. From diagnosis to enrolment, patients had developed a median of six (range: 1–55) complications; 19.6% were grade ≥3. The most represented complications were endocrine/metabolic/nutrition disorders (91.5%), surgical/medical procedures (67.7%) and blood/lymphatic system disorders (64.7%). Real‐world data generated by ULYSSES underscore the substantial complication burden of transfusion‐dependent β‐thalassaemia patients, routinely managed in Greece.

## 1 INTRODUCTION

Beta (β)‐thalassaemia is a hereditary blood disorder characterised by a deficiency of functional β‐globin chains [1, 2, 3, 4]. Based on severity, β‐thalassaemia has been traditionally classified as major, intermedia or minor, although a newer approach distinguishes patients into transfusion‐dependent, requiring lifelong regular blood transfusion for survival, and non‐transfusion‐dependent [5, 6]. The prevalence of β‐thalassaemia is high in Mediterranean countries and is estimated at about 25 per 100,000 population in Greece [7].

The pathophysiology of β‐thalassaemia is largely explained by the accumulation of excessive α‐globin chains due to their imbalance over β chains, which impairs the maturation and viability of the mature erythroid cells and their precursors, resulting in anaemia and related complications [2]. Management of β‐thalassaemia mainly aims at alleviating these states, with conventional therapies including red blood cell (RBC) transfusions, iron chelation therapy (ICT) and splenectomy, though the latter is gradually becoming obsolete [8, 9]. Such interventions have contributed to the substantial prolongation of patient life expectancy [10]. However, treatment approaches can also result in complications. The increased cumulative exposure to various treatment options that results from lifespan expansion of the thalassaemic patient, in combination with comorbidities related to advanced age, lead to an evolving spectrum of complications, with substantial impact on the affected individual and a burden to the healthcare system [11, 12].

The present study aimed to generate data on the patterns and determinants of disease‐ and treatment‐related complications in a representative sample of patients with transfusion‐dependent β‐thalassaemia treated in a routine care setting in Greece.

## 2 METHODS

### 2.1 Study design, setting, and population

ULYSSES (NIS‐GEN‐bTHAL‐001) was an epidemiological, multicentre, retrospective cross‐sectional cohort study, with a single‐visit primary data collection schedule. The study visit took place within the normal clinical practice setting, and study‐related information was collected through routine clinical/laboratory/imaging assessments, medical chart review and patient self‐report. Patients were enrolled over the planned recruitment period, from Oct 21, 2019 (first patient in) to Jun 15, 2020 (last patient in). Eligible patients were enrolled by haematology/thalassaemia specialists in 12 leading public hospital/university clinics covering all geographic regions of Greece, which provide care for about two‐thirds of thalassaemia patients in the country.

Eligible patients were adults with transfusion‐dependent β‐thalassaemia diagnosed ≥12 months before enrolment. Transfusion dependency was defined as the receipt of ≥6 RBC units (excluding transfusions for elective surgery) with no transfusion‐free period ≥35 days during the 24 weeks before enrolment. Patients with a diagnosis of haemoglobin S/β‐ or α‐thalassaemia, women who were pregnant during the previous year, recipients of bone marrow transplantation and patients who were concurrently participating in any interventional clinical trial were excluded. Participants provided signed informed consent.

The study was designed and conducted in accordance with the International Society for Pharmacoepidemiology Guidelines for Good Pharmacoepidemiology Practice, the Declaration of Helsinki and all applicable local rules and regulations. The study was approved by the Institutional Review Boards of all participating sites (Table SI).

### 2.2 Study objectives and outcomes

The primary aim of the study was to determine the prevalence and severity of disease‐ and treatment‐related complications in a representative sample of patients with transfusion‐dependent β‐thalassaemia, managed in routine care settings in Greece. The study also aimed to examine the prevalence and severity of complications in the age groups of 18–40 years and > 40 years. Additional objectives were to determine the prevalence of β‐thalassaemia phenotypic and genotypic forms, to depict current and past β‐thalassaemia management patterns and to capture the rate of hospitalisation due to disease‐ and treatment‐related complications during the 48‐week period prior to enrolment. Lastly, the study aimed to assess the anaemia, iron load status and RBC transfusion burden in the 24‐ or 48‐week periods prior to enrolment, as well as to explore the potential influence of patient and disease characteristics on the presence of complications.

Clinically significant medical/surgical history and comorbid conditions as well as disease‐related complications were coded using Medical Dictionary for Regulatory Activities (MedDRA) v.23.0 terminology, while the severity of complications was graded using the National Cancer Institute Common Terminology Criteria for Adverse Events (NCI CTCAE).

Study data were collected via web‐based electronic data capture (use of electronic case report form) fulfilling all data privacy rules.

### 2.3 Statistical approach

The sample size calculation was based on the study's primary endpoint. A sample of 200 patients was considered adequate, as it offered a maximum margin of error below 7.0% for the maximum indetermination of any complication frequency in the overall population (i.e., the estimation of a frequency of 50%, where the margin of error is largest). This represents a scientifically acceptable level of precision, and was thus sufficient for the primary endpoint, without the requirement to include the entire Greek registry of these patients. In order to control for and minimise selection bias, physicians were requested to consecutively enrol the first patients attending their clinic that met the eligibility criteria. Information bias, due to the involvement of several laboratories rather than a central laboratory, was minimised with the implementation of source data verification and quality assurance measures.

The normality of the distribution of continuous variables was examined using the Shapiro‐Wilk test, with data presented as mean (standard deviation, SD) when following a normal distribution and median (interquartile range, IQR) when not. No imputation for missing data was implemented, except for partial dates. In the context of the primary objective, the number and proportion of patients with at least one disease‐ and/or treatment‐related complication were calculated along with the relevant 95% Wald confidence intervals (CIs). The hospitalisation rate per patient‐year along with 95% CI during the 48‐week period prior to enrolment was estimated using a Poisson regression model. The association of the presence of disease‐ and/or treatment‐related complications was examined using univariable and multivariable logistic regression analysis. The multivariate logistic regression model was derived using a stepwise procedure based on the minimisation of Akaike's information criterion, while, due to separability issues, Firth's penalised likelihood approach of logistic regression was used. All statistical tests were two‐sided and at a 0.05 significance level. Statistical analysis and sample size determination were performed using SAS v9.4 (SAS Institute, Cary, NC).

## 3 RESULTS

### 3.1 Patient disposition and characteristics

A total of 208 patients were enrolled in the study; seven patients were excluded from the analysis, since they did not fulfil all eligibility criteria, resulting in 201 patients ultimately analysed.

Among eligible patients, 129 (64.2%) were female; the median (IQR) age at enrolment was 45.7 (40.2–50.5) years, with 49 (24.4%) patients aged 18–40 years and the remaining 152 (75.6%) aged > 40 years. Clinically significant medical/surgical history and comorbidities, excluding β‐thalassaemia‐ and treatment‐related complications were recorded for 115 patients at enrolment, with the most frequently listed in Table 1, along with other characteristics.

**TABLE 1:** Patient, disease and treatment characteristics.

Patient, disease and treatment characteristics	Value (*N* = 201)
Age, median (IQR), years	45.7 (40.2–50.5)
18–40 years, *n* (%)	49 (24.4)
>40 years, *n* (%)	152 (75.6)
Females, *n* (%)	129 (64.2)
Caucasian, *n* (%)	201 (100.0)
Employment status, *n* (%)	
Employed	77 (38.3)
Unemployed	11 (5.5)
Retired	76 (37.8)
Other	37 (18.4)
BMI classification, *n* (%)	
Underweight (<18.5 kg/m^2^)	3 (1.5)
Normal weight (18.5 kg/m^2^≤ to <25 kg/m^2^)	138 (68.7)
Overweight (25 kg/m^2^≤ to <30 kg/m^2^)	46 (22.9)
Obese (≥30 kg/m^2^)	14 (7.0)
Non‐thalassaemia‐related clinically significant medical/surgical history/comorbidities, *n* (%)	115 (57.2)
Most common medical conditions/comorbidities, *n* (%)	
Appendicectomy	21 (10.4)
Cholecystectomy	12 (6.0)
Caesarean section	8 (4.0)
Pneumonia	8 (4.0)
Age at β‐thalassaemia diagnosis, median (IQR), years	0.8 (0.4–2.8)
Time elapsed from β‐thalassaemia diagnosis to enrolment, mean (SD), years	42.9 (7.8)
β‐thalassaemia clinical phenotype, *n* (%)	
β‐thalassaemia major	170 (84.6)
β‐thalassaemia intermedia	31 (15.4)
Family history of β‐thalassemia major or intermedia, *n* (%)	43 (21.4)
Age at RBC transfusion initiation, median (IQR), years	1.3 (1.0–5.0)
Receipt of ICT at enrolment, *n* (%)	201 (100.0)
Age at ICT initiation, median (IQR), years	6.4 (3.2–12.9)
Exposure to ICT from diagnosis to enrolment, median (IQR), years	35.6 (30.0–40.0)
History of splenectomy, *n* (%)	107 (53.2)
Age at splenectomy, median (IQR), years	22.0 (14.0–29.0)
Other therapies, *n* (%)	107 (53.2)
Folic acid supplements	97 (48.3)
Acetylsalicylic acid	26 (12.9)
Chemotherapeutic agents (e.g., hydroxyurea)	9 (4.5)
Vitamin C supplements	2 (1.0)

Abbreviations: BMI, body mass index; ICT, iron chelation therapy; IQR, interquartile range; RBC, red blood cell.

Parameters relevant to patients’ anaemia and iron overload status over the 48‐week and 24‐week periods prior to enrolment or based on the most recent available measurement are displayed in Table 2.

**TABLE 2:** Anaemia and iron overload status prior to study enrolment.

Blood or liver measurement	Value (*N* = 201)
48‐week period prior to enrolment	
Average pre‐transfusion haemoglobin, median (IQR), g/dL	9.9 (9.4–10.3)
Average pre‐transfusion haematocrit, median (IQR), % L/L^a^	29.9 (28.6–31.4)
Average serum ferritin, median (IQR), μg/L^b^	549.3 (287.2–1034.8)
24‐week period prior to enrolment	
Average pre‐transfusion haemoglobin, median (IQR), g/dL	10.0 (9.4–10.4)
Average pre‐transfusion haematocrit, median (IQR), % L/L^c^	30.0 (28.6–31.3)
Average serum ferritin, median (IQR), μg/L^d^	540.3 (281.9–990.0)
Average serum ferritin categories, *n* (%)	
<1000 μg/L	141 (70.1)
1000–2500 μg/L	29 (14.4)
>2500 μg/L	17 (8.5)
Missing	14 (7.0)
Most recent available	
Iron overload grade based on most recent LIC measurement, *n* (%)	
Normal (<3.5 mg Fe/g dw)	132 (65.7)
Mild (≥3.5 to <7.0 mg Fe/g dw)	27 (13.4)
Moderate (≥7.0 to ≤15.0 mg Fe/g dw)	27 (13.4)
Severe (>15.0 mg Fe/g dw)	10 (5.0)
Missing	5 (2.5)
MRI LIC, median (IQR), mg Fe/g dw^e^	2.2 (1.3–4.8)
Myocardial T2*, median (IQR), ms^e^	36.8 (32.6–39.3)
Myocardial iron overload grade based on most recent MRI T2*, *n* (%)	
Normal (>20 ms)	182 (90.5)
Mild (≥14 to ≤20 ms)	5 (2.5)
Moderate (≥10 to <14 ms)	4 (2.0)
Severe (<10 ms)	5 (2.5)
Missing	5 (2.5)

Abbreviations: dw, dry weight; IQR, interquartile range; LIC, liver iron concentration; MRI, magnetic resonance imaging; T2*, elevated transverse relaxation time.

^a^ Two missing.

^b^ One missing.

^c^ Three missing.

^d^ Fourteen missing.

^e^ Five missing.

### 3.2 Β‐thalassaemia disease characteristics

Patients’ median (IQR) age at β‐thalassaemia diagnosis was 0.8 (0.4–2.8) years, with a mean (SD) of 42.9 (7.8) years elapsed from diagnosis to enrolment. The β‐thalassaemia clinical phenotype, assessed by the physicians was characterized as major in 170 (84.6%) patients and intermedia in the remaining 31 (15.4%) (see Table SII). All patients referred to as thalassemia intermedia were transfusion independent at the time of the study but became transfusion dependent in older age.

**FIGURE 1:**
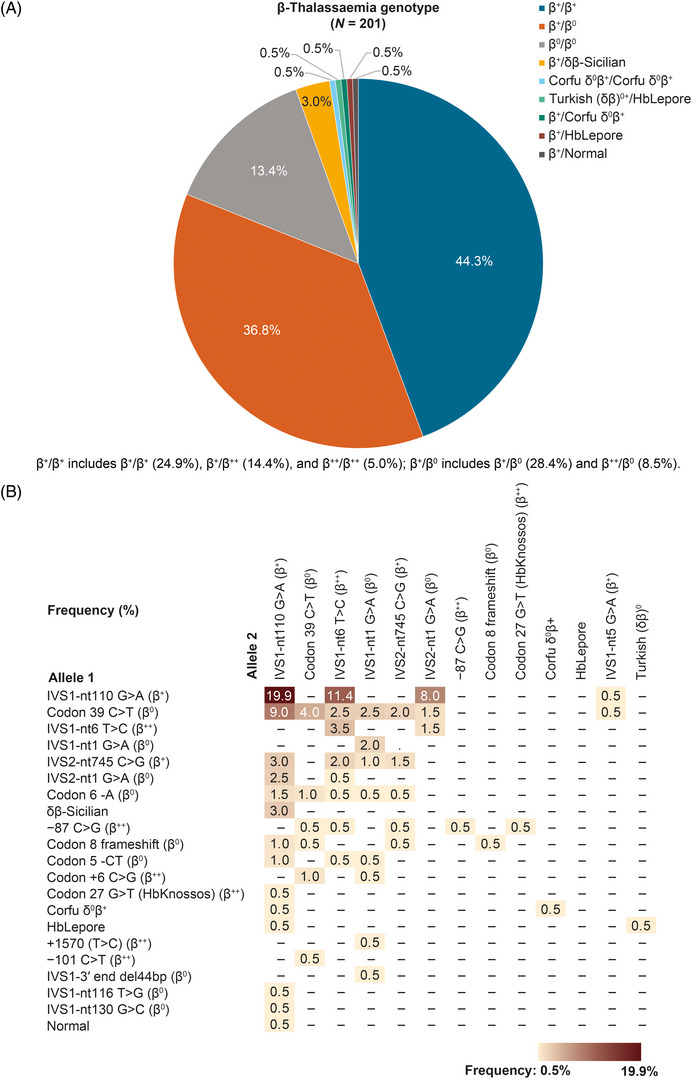
Genotypic profile of the study patient population. (A) Distribution of patients by β‐thalassemia genotype. (B) Frequency of β‐globin gene mutations in the study population.

The genetic profile of the patient population is displayed in Figure 1, with Figure 1A showing the distribution of β‐globin alleles and Figure 1B the frequency of the specific β‐globin mutations. The two most frequently‐represented genotypes were β^+^β^0^ (*n =* 57, 28.4%) and β^+^β^+^ (*n =* 50, 24.9%), while the most prevalent mutations in the study population were IVS1‐nt110 G > A (*n =* 128, 63.7%), followed by codon 39 C > T (*n =* 51, 25.4%) and IVS1‐nt6 T > C (*n =* 46, 22.9%). In addition to the β‐globin gene mutation, 18 (9.0%) patients also carried a mutation, deletion and/or multiplication in one or two α‐globin genes, among which two patients carried a triplication of the α‐globin gene.

A family history of β‐thalassaemia major or intermedia in first‐ or second‐degree relatives was reported by 43 (21.4%) patients.

### 3.3 Current and past β‐thalassaemia management patterns

According to the study inclusion criteria, all patients were transfusion dependent, with the median (IQR) age at receipt of the first transfusion being 1.3 (1.0–5.0) years. The median (IQR) number of transfusions per patient over the 48‐week period prior to enrolment was 23.0 (18.0–28.0), corresponding to a mean (SD) total number of 34.2 (9.5) RBC units.

In addition to RBC transfusions prior to enrolment, all patients (100.0%) had received ICT, 107 (53.2%) had undergone splenectomy and 107 also (53.2%) had received other kinds of treatment (mainly folic acid supplements) (Tables 1 and SIII). The proportions of patients who had undergone splenectomy were 28.6% (14/49) and 61.2% (93/152) in the age groups of 18–40 years and > 40 years, respectively.

### 3.4 Β‐thalassaemia disease‐ and treatment‐related complications

At enrolment, medical records were available for a mean percentage of 93.2% of the time since diagnosis, with the full disease‐ and treatment‐related medical history available for 158 (78.6%) patients and partial relevant information for the remaining 43 (21.4%) patients.

**FIGURE 2:**
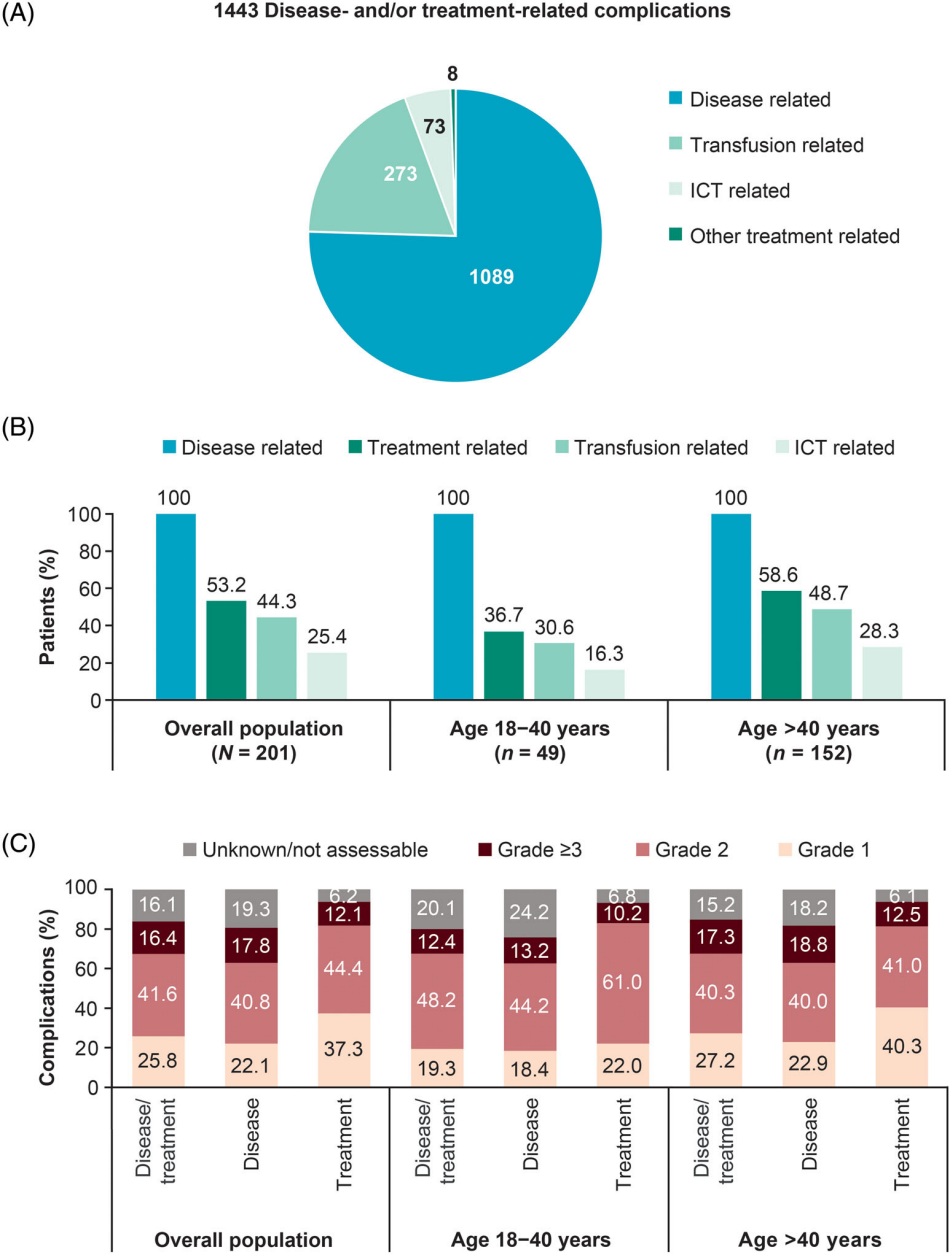
History of complications in the study population. (A) Number of disease and treatment complications between diagnosis and enrolment in the overall population. (B) Percentage of patients with disease and treatment complications between diagnosis and enrolment overall and by age group. Patients who had complications attributed to more than one factor have been included in all applicable categories. (C) Severity of disease and treatment complications overall and by age group. ICT, iron chelation therapy.

During the period between diagnosis and enrolment, at least one complication related to β‐thalassaemia itself was recorded for all patients, while at least one complication related to the treatment of β‐thalassaemia was recorded for 53.2% (*n =* 107, 95% CI: 46.3–60.1) (see Table SIV). A total of 1443 disease‐ and/or treatment‐related complications were recorded (median 6; range: 1–55 per patient) over this period (Figure 2A). The median number of disease‐related complications per patient was five (range: 1–21), and that of treatment‐related complications was one (range: 0–35).

**FIGURE 3:**
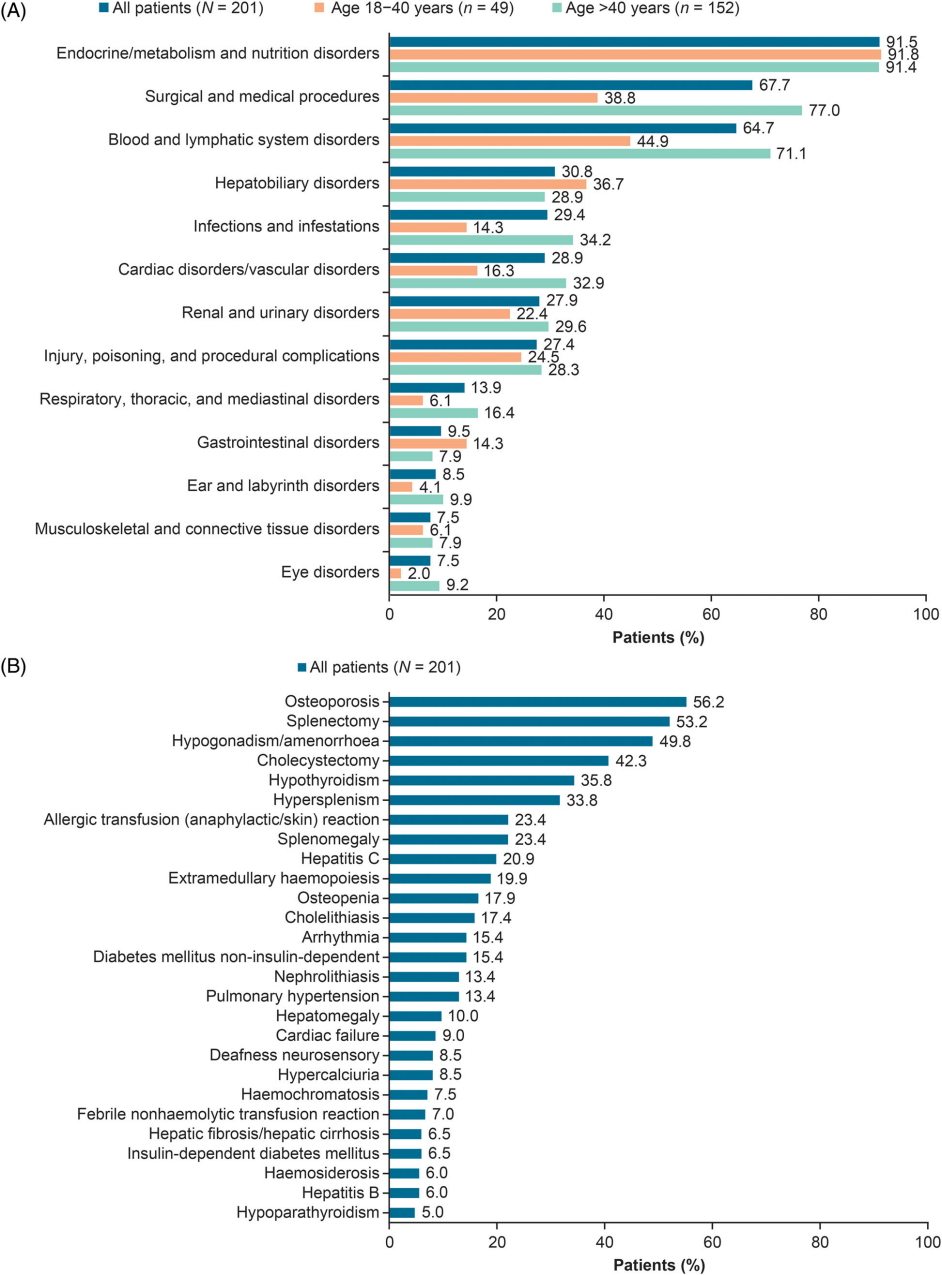
Most common β‐thalassemia disease‐ and treatment‐related complications in the overall population. (A) Most common complications by System Organ Class in the overall study population, and in the subgroups of 18–40 and > 40 years of age. (B) Complications in ≥5.0% of the overall study population.

**FIGURE 4:**
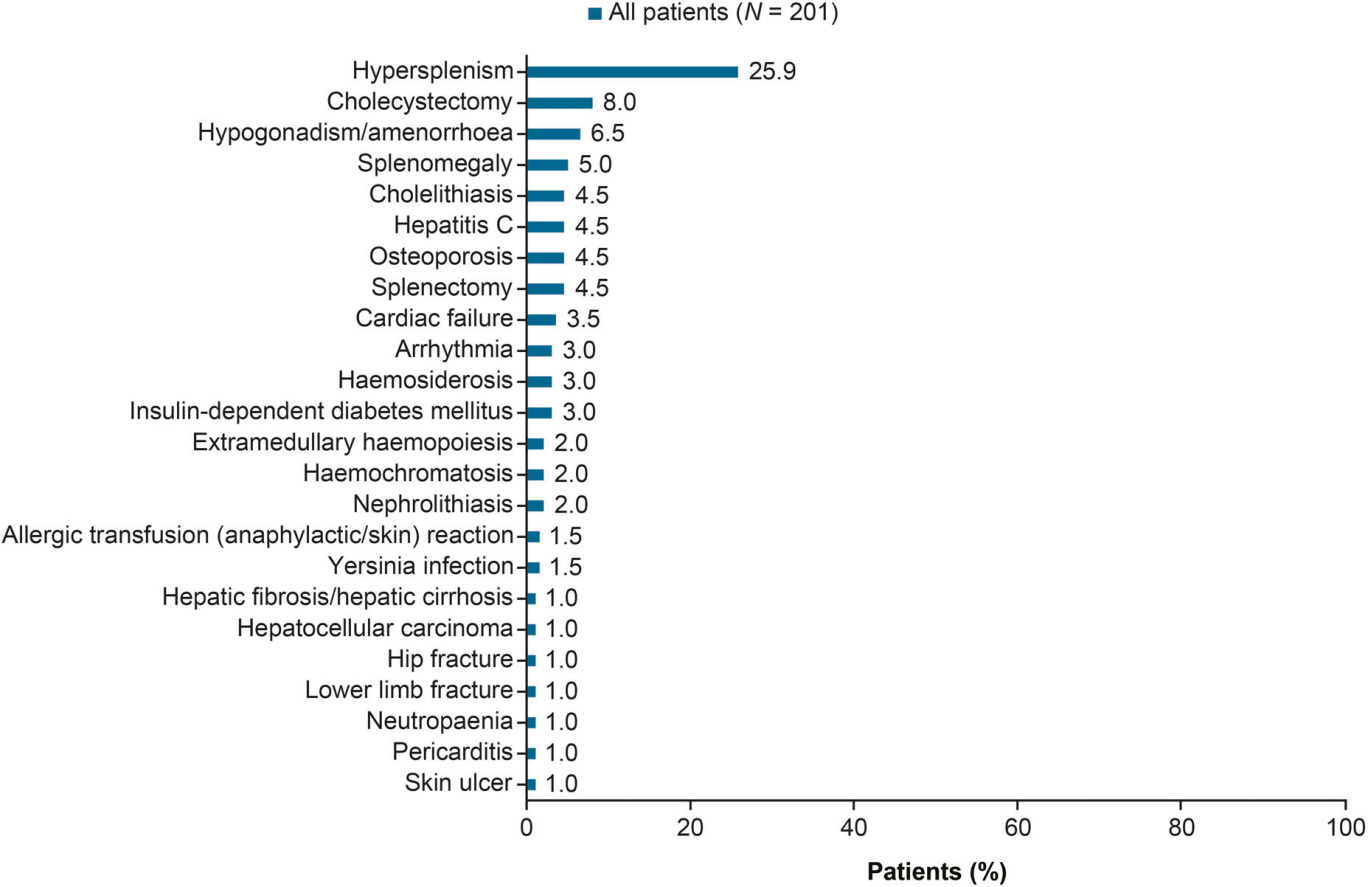
Most common grade ≥3 complications in the overall study population.

In the age group of 18–40 years, 18 patients [36.7%; 50.0% (7/14) of splenectomized and 31.4% (11/35) of non‐splenectomized patients] experienced at least one treatment‐related complication, while in the age group of >40 years, 89 patients [58.6%; 60.2% (56/93) of splenectomized and 55.9% (33/59) of non‐splenectomized patients] experienced such complications (Figure 2B,C). The most common disease‐ and treatment‐related complications are listed in Figure 3 and Figure S1 for the overall population and by age group. Grade ≥3 complications are presented in Figure 4 for the overall population. The complete breakdown of disease‐ and treatment‐related complications is presented in Table SV.

During the 48‐week period prior to enrolment, apart from transfusion sessions, 14 hospitalisations due to complications were recorded in the overall eligible population, all in non‐intensive care units, yielding a hospitalisation rate of 0.076 per patient‐year (95% CI: 0.045–0.128). The mean (SD) length of stay in the hospital was 5.6 (4.2) days.

### 3.5 Association of patient and disease characteristics with β‐thalassaemia disease‐ and treatment‐related complications at enrolment

**FIGURE 5:**
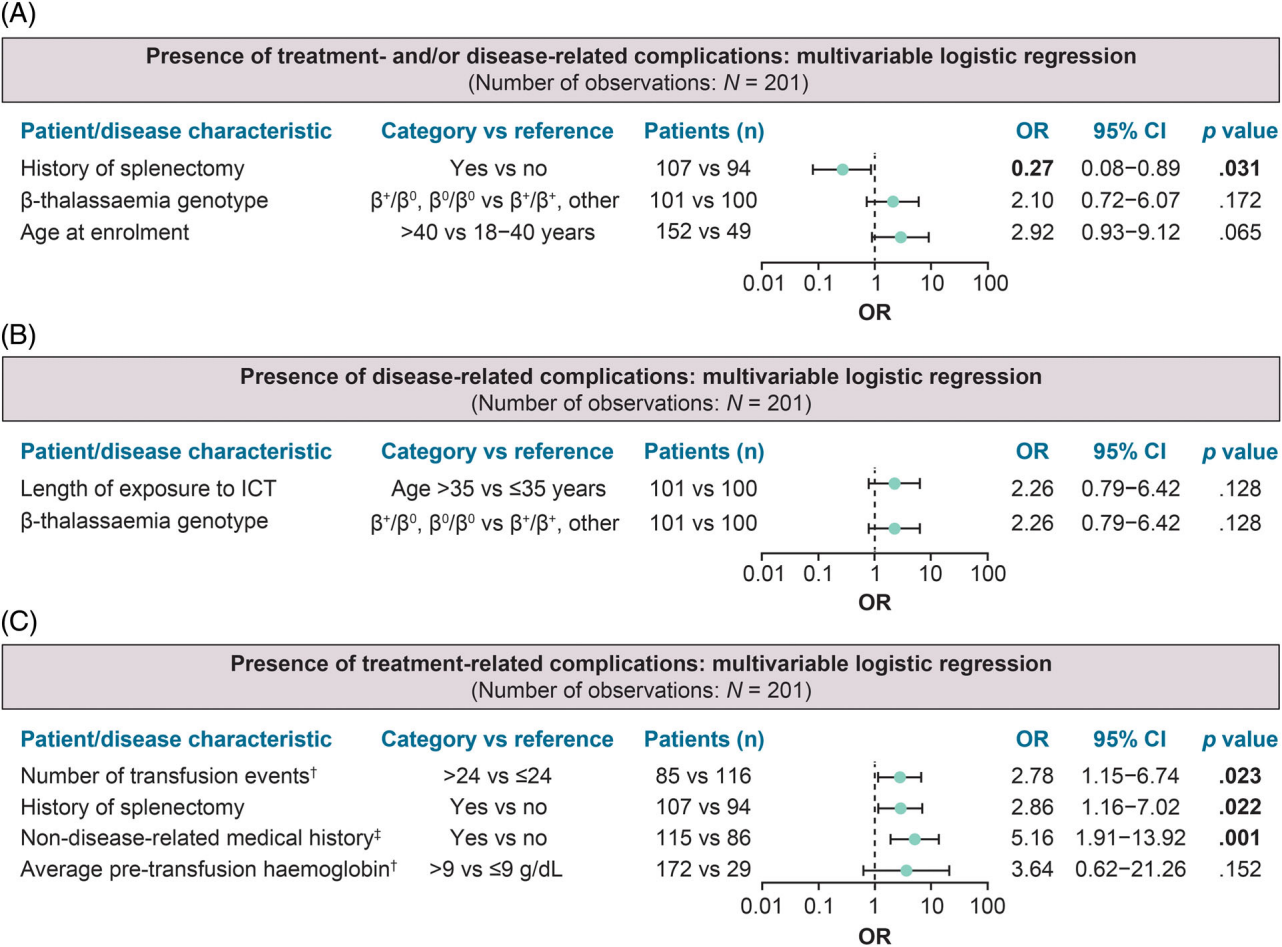
Association of patient and disease characteristics with the presence of β‐thalassemia disease‐ and treatment‐related complications at the study visit. (A) Multivariable logistic regression analysis for the association of patient and disease characteristics with the presence of disease‐ and/or treatment‐related complications. (B) Multivariable logistic regression analysis for the association of patient and disease characteristics with the presence of disease‐related complications. (C) Multivariable logistic regression analysis for the association of patient and disease characteristics with the presence of treatment‐related complications. Values in bold indicate statistical significance, that is, *p* < 0.05. ^a^In the 48 weeks before enrolment; ^b^Includes surgical and comorbidity history. CI, confidence interval; ICT, iron chelation therapy; OR, odds ratio.

The potential association of selected patients and disease characteristics with the presence of the most prevalent treatment‐ and disease‐related complications at enrolment was investigated by univariable analysis (Table SVI) and multivariable logistic regression analyses, to exclude variables associated with other variables (Figure 5). By multivariable analyses, splenectomized patients had an approximately 3‐fold higher odds ratio of having at least one treatment‐related complication compared with those not splenectomised (Figure 5C). Overall, splenectomised patients had a lower probability of the manifestation of complications (Figure 5A). Moreover, patients with clinically significant medical/surgical history/comorbidities not related to β‐thalassaemia and patients having received >24 transfusion sessions in the 48 weeks prior to enrolment had an approximately 5‐fold and 3‐fold higher odds ratio of having at least one β‐thalassaemia treatment‐related complication at enrolment, than the other patients, respectively.

## 4 DISCUSSION

ULYSSES provides epidemiological data on the profile and complication burden of transfusion‐dependent β‐thalassaemia patients, managed in routine care in Greece. Over the 48‐week pre‐enrolment period, the average pre‐transfusion haemoglobin was 9.9 g/dL, within recommended 9.5–10.5 g/L target [13]; average serum ferritin in ULYSSES was 549.3 μg/L, median liver iron concentration (LIC) was 2.2 mg Fe/g dry weight (dw) and median elevated transverse relaxation time (T2*), which serves as a measure to assess myocardial iron deposition and the risk for cardiac complications [17], was 36.8 ms, with 92.9% of evaluable patients having normal myocardial iron levels (> 20 ms). Maintenance of serum ferritin below 1000 μg/L has been associated with improved clinical outcomes among all patients [18]; LIC up to 7 mg/g dw generally has no apparent adverse effects, while sustained LIC above 15–20 mg/g dw is linked with worsening prognosis [19, 20] and abnormalities [21]. Thus, patients in ULYSSES seem to have rather adequately controlled iron levels, compared with other previously published observational studies [14, 15, 16, 22, 23, 24, 25, 26, 27, 28, 29, 30, 31]. Several factors could contribute to the observed differences, including variability in patient populations, genetic background, regions and observation periods, which determine available resources and application of different treatment modalities.

The complications recorded in ULYSSES are those most encountered among transfusion‐dependent thalassaemia patients [32]. The most represented complications affecting at least one‐third of the patients in ULYSSES were endocrine disorders/metabolic/nutrition disorders (such as osteoporosis; 56.2%), surgical/medical procedures and blood/lymphatic system disorders (such as hypersplenism; 33.8%), which were within the ranges recorded in other studies [8, 15, 32, 33, 34, 35, 36, 37]. The most frequently reported surgical procedure in ULYSSES was splenectomy (53.2% of patients). Splenectomy rates in other cohorts range from 40% to 70%, with higher prevalence among older patients [14, 22, 38, 39], consistent with our data. Cardiac/vascular disorders are among the most important life‐threatening complications in β‐thalassaemia [40] and were reported for 29% of the patients in ULYSSES. Furthermore, 9% of patients experienced heart failure, consistent with the 9% pooled prevalence estimated in a meta‐analysis of data from 10,968 patients, with transfusion‐dependent β‐thalassaemia major from multiple continents [41].

A major concern for transfusion‐dependent patients is the risk of infections, with hepatitis C being the most common. In ULYSSES, hepatitis C infection was recorded for 20.9% of the overall population. Differences between age groups (25.7% in > 40 years versus 6.1% in 18–40 years) are presumably attributed to the improvement of blood screening protocols. These rates are within the 5.9%–28.3% range, reported in a systematic review of studies in transfusion‐dependent thalassaemia with enrolment in 2005 or later [22, 38, 39, 42, 43]. Three patients in the present study were diagnosed with hepatocellular carcinoma, a prevalence within the previously reported range of 0.75%‐3.5% among patients with thalassaemia major [22, 44, 45].

Although not statistically compared, the proportion of patients with treatment‐related complications was higher in the > 40 age group, than in the 18–40 age group. This was expected, since advancing age, as well as the cumulative effect of longer exposure to treatments, may contribute to the complication burden. Multivariable analysis showed that disease unrelated medical history/comorbidities, prior splenectomy and a higher number of transfusions were associated with a higher odds ratio of having experienced a treatment‐related complication at enrolment. In contrast, in univariable analysis, splenectomy was associated with a lower odds ratio of having a hepatobiliary disorder, which may also partially explain why complications from this system were more common among younger patients.

The main limitations of this study are attributed to its retrospective observational design. At enrolment, for 21.4% of the patients, partial medical records were available; thus, transient complications may have been under‐reported. This bias is expected to be small, as medical records were available for 93.2% of the time since diagnosis in the overall population. Regarding external validity, every effort was made for the study population to be representative of β‐thalassaemia patients treated in Greece by enrolling patients with a non‐limiting set of clinical characteristics from geographically diverse regions across Greece. All the major thalassemia centres in Greece participated in the study. Patients followed in smaller centres/units were not included, but as they are a minority, it is highly unlikely that their data were significantly different from the ones reported in the study.

The results of the present real‐world study underscore a considerable complication burden of transfusion‐dependent β‐thalassaemia and the need for effective monitoring and improved therapies for these patients.

## AUTHOR CONTRIBUTIONS

Conception and design: AKa, EV, KK‐M, and DT

Data acquisition: AKa, EV, SD, EKl, IL, FP, MDD, SL, LE, EKa, and AKo

Data analysis: PV

Data interpretation: AKa, EV, CD, PV, and AKo

Manuscript writing: All authors

Final approval of manuscript: All authors

## CONFLICT OF INTEREST STATEMENT

Antonis Kattamis has received grants from Novartis and Bristol Myers Squibb/Celgene; consulting fees from Agios Pharmaceuticals, Amgen, Bristol Myers Squibb/Celgene, Crispr/Vertex, Ionis Pharmaceuticals, Novartis and Vifor; honoraria from Novartis, Bristol Myers Squibb/Celgene, Chiesi and Crispr/Vertex; and meeting/travel support from Bristol Myers Squibb/Celgene.

Sophia Delicou has received consulting fees, honoraria and meeting/travel support from Novartis and Bristol Myers Squibb.

Foteini Petropoulou has received grants and/or study funding from Genesis Pharma, Protagonist Therapeutics and Celgene; consulting fees from Bristol Myers Squibb; and has attended advisory board meetings for Bristol Myers Squibb.

Michael D. Diamantidis has received study funding/grants from Genesis Pharma, Ionis Pharmaceuticals, Novartis, Forma Therapeutics Inc., Synteract and Vifor International Inc.; honoraria from Genesis Pharma, Uni‐Pharma, Bristol Myers Squibb and Abbvie; meeting/travel support from Demo Company, Bristol Myers Squibb and Genesis Pharma; and has attended advisory board meetings for Bristol Myers Squibb.

Loukia Evliati has received study funding from Genesis Pharma.

Panagiotis Viktoratos is an employee of Bristol Myers Squibb.

Alexandra Kourakli has received study funding/grants from Bristol Myers Squibb; consulting fees from Forma Therapeutics Inc.; honoraria from Novartis, Bristol Myers Squibb and Elpen; meeting/travel support from Glaxo and Rafarm; and has attended advisory board meetings for Genesis, Gilead and Agios.

Ersi Voskaridou, Evangelos Klironomos, Ioannis Lafiatis, Stylianos Lafioniatis, Eleni Kapsali, Kiki Karvounis‐Marolachakis, Despoina Timotheatou and Chrysoula Deligianni have no disclosures to declare.

## Supporting information



Supporting Information

## Data Availability

The Bristol Myers Squibb policy on data sharing may be found at: https://www.bms.com/researchers‐and‐partners/independent‐research/data‐sharing‐request‐process.html.
