# Supplementary material for: Real‐world complication burden and disease management paradigms in transfusion‐related β‐thalassaemia in Greece: Results from ULYSSES, an epidemiological, multicentre, retrospective cross‐sectional study
Source: EJHaem. 2023 May 23;4(3):569–81. doi: 10.1002/jha2.695 (PMC10435690; doi:10.1002/jha2.695)
Supplement: Supplementary file 1 — Supporting Information [file JHA2-4-569-s001.docx]

**Supplement Data**

**Fig S1.** β-thalassemia disease- and treatment-related complications by MedDRA preferred term in the age groups of 18–40 and >40 years. (A) Complications in ≥5.0% of patients 18–40 years of age. (B) Complications in ≥5.0% of patients >40 years of age. MedDRA, Medical Dictionary for Regulatory Activities.


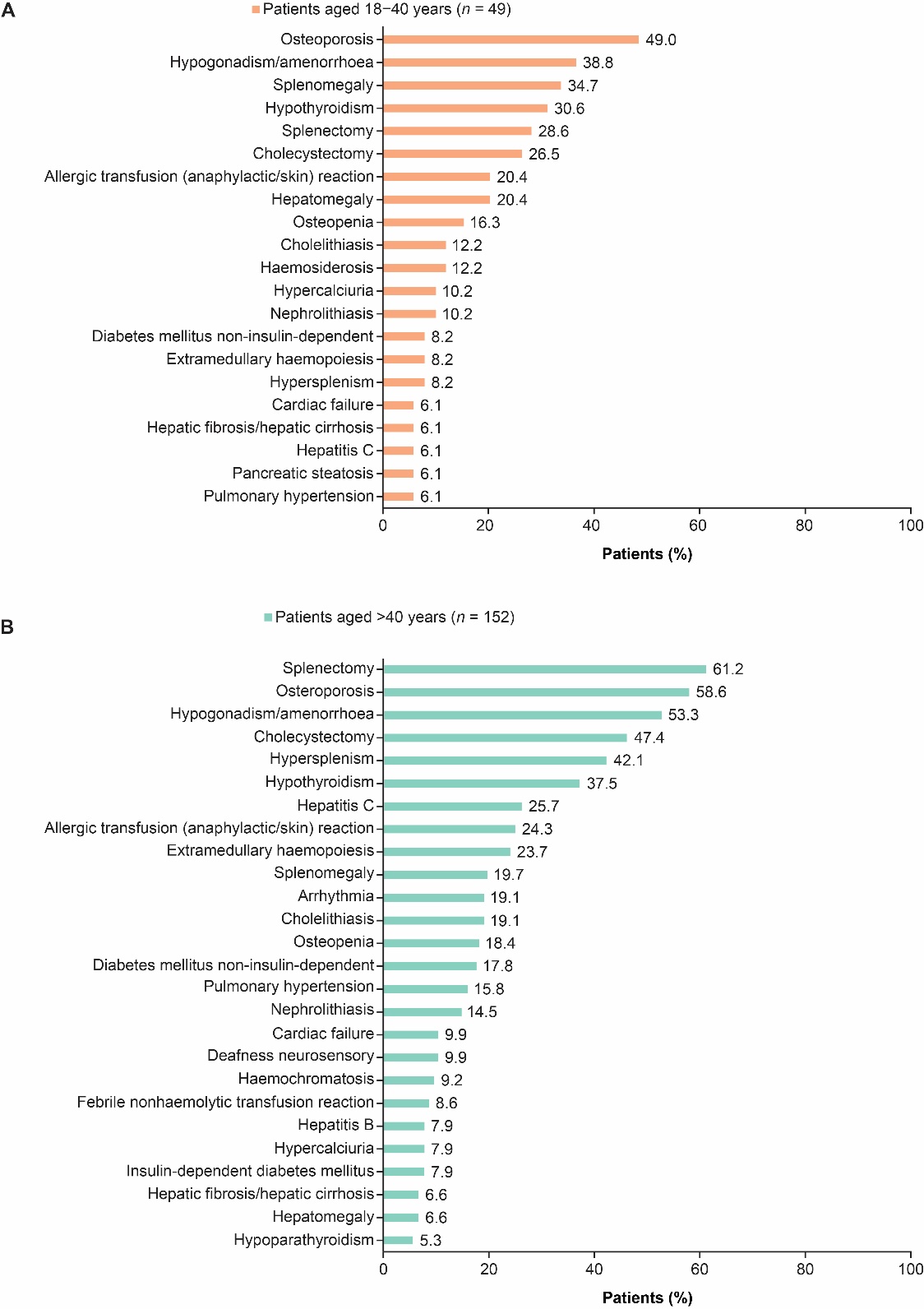


**Table SI.** Information on IRB approvals

| Principal Investigator | Affiliation | City/region | IRB approval date |
| --- | --- | --- | --- |
| Delicou Sofia | General Hospital of Athens “Ippokratio” | Athens/Attica | 23-Jul-2019 |
| Diamantidis Michael | General Hospital of Larissa  “Koutlimpaneio - Triantafyllio” | Larissa/Thessaly | 07-Jun-2019 |
| Eftichiadis Eftichios | General Hospital of Karditsa | Karditsa/Thessaly | 20-Jun-2019 |
| Evliati Loukia | General Hospital of Athens  “Evaggelismos” | Athens/Attica | 18-Jul-2019 |
| Vlachaki Efthymia | General Hospital of Thessaloniki  “Ippokratio” | Thessaloniki/Central Macedonia | NA |
| Kapsali Eleni | University General Hospital of Ioannina | Ioannina/Epirus | 12-Sep-2019 |
| Katsatou Marianna | General Hospital of Corfu “Agia Eirini” | Corfu/Ionian Islands | 15-Jul-2019 |
| Kattamis Antonios | Children's Hospital of Athens “Aghia Sophia” | Athens/Attica | 11-Oct-2019 |
| Klironomos Evangelos | General Hospital of Heraklion“Venizeleio” | Heraklion/Crete | 26-Jun-2019 |
| Kourakli Alexandra | University Hospital of Patras | Rion/Western Greece | 30-Jul-2019 |
| Lafiatis Ioannis | General Hospital of Mytilene  “Vostaneio” | Mytilene/North Aegean | 12-Jun-2019 |
| Lafioniatis Stylianos | General Hospital of Volos “Achilopoulio” | Volos/Thessaly | 29-Aug-2019 |
| Pantelidou Despoina | University General Hospital of Thessaloniki “AHEPA” | Thessaloniki/Central Macedonia | NA |
| Petropoulou Foteini | General Hospital of Athens  “G Gennimatas” | Athens/Attica | 08-Jul-2019 |
| Voskaridou Ersi | General Hospital of Athens “Laiko” | Athens/Attica | 26-Jul-2019 |

IRB, Institutional Review Board; NA, not applicable – these site did not obtain IRB approval and did not enrol patients.

**Table SII.** Distribution of β-globin genotypes per β-thalassaemia clinical phenotype

|  |  | | Phenotype | |
| --- | --- | --- | --- | --- |
|  | Overall  (*N* = 201) | β-thalassaemia  major (*n* = 170) | | β-thalassaemia  intermedia (*n* = 31) |
| Genotype^a^ | *n* (%) | *n* (%) | | *n* (%) |
| β^+^/β^0^ | 57 (28.4) | 53 (31.2) | | 4 (12.9) |
| β^+^/β^+^ | 50 (24.9) | 45 (26.5) | | 5 (16.1) |
| β^++^/β^+^ | 29 (14.4) | 25 (14.7) | | 4 (12.9) |
| β^0^/β^0^ | 27 (13.4) | 24 (14.1) | | 3 (9.7) |
| β^++^/β^0^ | 17 (8.5) | 13 (7.6) | | 4 (12.9) |
| β^++^/β^++^ | 10 (5.0) | 3 (1.8) | | 7 (22.6) |
| Other | 11 (5.5) | 7 (4.1) | | 4 (12.9) |
| β^+^/δβ-Sicilian | 6 (3.0) | 3 (1.8) | | 3 (9.7) |
| Corfu δβ/Corfu δβ | 1 (0.5) | 1 (0.6) | | – |
| Turkish (δβ)^0^/HbLepore | 1 (0.5) | – | | 1 (3.2) |
| β^+^/Corfu δβ | 1 (0.5) | 1 (0.6) | | – |
| β^+^/HbLepore | 1 (0.5) | 1 (0.6) | | – |
| β^+^/Normal | 1 (0.5) | 1 (0.6) | | – |

^a^Genotypes have been derived from the gene-mutation profile of each patient.

**Table SIII.** β-thalassaemia management patterns (other than transfusion) since diagnosis

|  | Value (*N* = 201) |
| --- | --- |
|  | *n* (%) |
| ICT | 47 (23.4) |
| ICT + |  |
| Splenectomy | 47 (23.4) |
| Folic acid supplements | 43 (21.4) |
| Folic acid supplements, splenectomy | 29 (14.4) |
| Folic acid supplements, acetylsalicylic acid, splenectomy | 14 (7.0) |
| Acetylsalicylic acid, splenectomy | 8 (4.0) |
| Folic acid supplements, hydroxyurea, splenectomy  SplSple | 4 (2.0) |
| Folic acid supplements, hydroxyurea  SplSple | 2 (1.0) |
| Folic acid supplements, hydroxyurea, acetylsalicylic acid, splenectomy | 2 (1.0) |
| Folic acid supplements, vitamin C supplements, splenectomy | 2 (1.0) |
| Acetylsalicylic acid | 1 (0.5) |
| Hydroxyurea, splenectomy | 1 (0.5) |
| Folic acid supplements, acetylsalicylic acid | 1 (0.5) |

ICT, iron chelation therapy.

**Table SIV.** History of treatment- and disease-related complications in the overall eligible population

|  |  | | 95% Wald CI | |
| --- | --- | --- | --- | --- |
| Disease- and treatment-related complications in the overall eligible population; *N* = 201 |  | *n* (%) | Lower limit | Upper limit |
| Patients with at least one treatment- and/or disease-related complication | Yes | 201 (100.0) | – | – |
| At least one complication related to β-thalassaemia | Yes | 201 (100.0) | – | – |
| At least one treatment-related complication | No | 94 (46.8) | 39.9 | 53.7 |
|  | Yes | 107 (53.2) | 46.3 | 60.1 |
| Related to transfusions | No | 112 (55.7) | 48.9 | 62.6 |
|  | Yes | 89 (44.3) | 37.4 | 51.1 |
| Related to ICT | No | 150 (74.6) | 68.6 | 80.6 |
|  | Yes | 51 (25.4) | 19.4 | 31.4 |
| Related to other β-thalassaemia therapy excluding transfusions and ICT | No  Yes | 197 (98.0)  4 (2.0) | 96.1  0.1 | 99.9  3.9 |

CI, confidence interval; ICT, iron chelation therapy.

**Table SV.** β-thalassaemia disease- and treatment-related complications in the overall eligible population

| Disease- and treatment-related complications in the overall eligible population; *N* = 201 | | | | | | | | | | |
| --- | --- | --- | --- | --- | --- | --- | --- | --- | --- | --- |
|  | Related to | | | | | | | | | |
| MedDRA v.23.0 SOC | Disease | | Transfusions | | | ICT | | | β-thalassaemia therapy other than transfusion/ICT | |
| MedDRA v.23.0 PT | n_ev_ | *n* (%) | n_ev_ | *n* (%) | n_ev_ | | *n* (%) | n_ev_ | | *n* (%) |
| Overall | 1089 | 201  (100.0%) | 273 | 89  (44.3%) | 73 | | 51  (25.4%) | 8 | | 4  (2.0%) |
| Endocrine disorders/metabolism and nutrition disorders | 409 | 184 (91.5%) | 10 | 9 (4.5%) | . | | . | 1 | | 1 (0.5%) |
| Osteoporosis | 113 | 113 (56.2%) | . | . | . | | . | . | | . |
| Hypogonadism/Amenorrhoea | 100 | 100 (49.8%) | . | . | . | | . | . | | . |
| Hypothyroidism | 72 | 72 (35.8%) | . | . | . | | . | . | | . |
| Osteopenia | 36 | 36 (17.9%) | . | . | . | | . | . | | . |
| Diabetes mellitus non-insulin-dependent | 31 | 31 (15.4%) | . | . | . | | . | . | | . |
| Haemochromatosis | 14 | 14 (7.0%) | 1 | 1 (0.5%) | . | | . | . | | . |
| Haemosiderosis | 5 | 4 (2.0%) | 9 | 8 (4.0%) | . | | . | . | | . |
| Insulin-dependent diabetes mellitus (LLT) | 13 | 13 (6.5%) | . | . | . | | . | . | | . |
| Hypoparathyroidism | 10 | 10 (5.0%) | . | . | . | | . | . | | . |
| Pancreatic steatosis | 5 | 5 (2.5%) | . | . | . | | . | . | | . |
| Hypocalcaemia | 1 | 1 (0.5%) | . | . | . | | . | 1 | | 1 (0.5%) |
| Metabolic disorder | 2 | 2 (1.0%) | . | . | . | | . | . | | . |
| Adrenal insufficiency | 1 | 1 (0.5%) | . | . | . | | . | . | | . |
| Glucose metabolism disorder | 1 | 1 (0.5%) | . | . | . | | . | . | | . |
| Goitre | 1 | 1 (0.5%) | . | . | . | | . | . | | . |
| Hyperthyroidism | 1 | 1 (0.5%) | . | . | . | | . | . | | . |
| Insulin-requiring type 2 diabetes mellitus | 1 | 1 (0.5%) | . | . | . | | . | . | | . |
| Thyroid mass | 1 | 1 (0.5%) | . | . | . | | . | . | | . |
| Thyroiditis | 1 | 1 (0.5%) | . | . | . | | . | . | | . |
| Surgical and medical procedures | 197 | 136 (67.7%) | 1 | 1 (0.5%) | . | | . | 1 | | 1 (0.5%) |
| Splenectomy | 106 | 106 (52.7%) | 1 | 1 (0.5%) | . | | . | . | | . |
| Cholecystectomy | 85 | 85 (42.3%) | . | . | . | | . | . | | . |
| Lithotripsy | 2 | 1 (0.5%) | . | . | . | | . | . | | . |
| Abortion induced | 1 | 1 (0.5%) | . | . | . | | . | . | | . |
| Hip arthroplasty | 1 | 1 (0.5%) | . | . | . | | . | . | | . |
| Internal fixation of fracture | 1 | 1 (0.5%) | . | . | . | | . | . | | . |
| Thyroidectomy | 1 | 1 (0.5%) | . | . | . | | . | . | | . |
| Ureteral stent insertion | . | . | . | . | . | | . | 1 | | 1 (0.5%) |
| Blood and lymphatic system disorders | 159 | 124 (61.7%) | 17 | 10 (5.0%) | 7 | | 7 (3.5%) | . | | . |
| Hypersplenism | 68 | 68 (33.8%) | . | . | . | | . | . | | . |
| Splenomegaly | 47 | 47 (23.4%) | . | . | . | | . | . | | . |
| Extramedulla ry haemopoiesis | 40 | 40 (19.9%) | . | . | . | | . | . | | . |
| Extravascular haemolysis | . | . | 10 | 8 (4.0%) | . | | . | . | | . |
| Alloimmunisation | . | . | 4 | 4 (2.0%) | . | | . | . | | . |
| Neutropenia | . | . | . | . | 4 | | 4 (2.0%) | . | | . |
| Autoimmune haemolytic anaemia | 1 | 1 (0.5%) | 1 | 1 (0.5%) | . | | . | . | | . |
| Hypercoagulation | 2 | 2 (1.0%) | . | . | . | | . | . | | . |
| Intravascular haemolysis | . | . | 2 | 2 (1.0%) | . | | . | . | | . |
| Agranulocytosis | . | . | . | . | 1 | | 1 (0.5%) | . | | . |
| Haemolysis | 1 | 1 (0.5%) | . | . | . | | . | . | | . |
| Leucocytosis | . | . | . | . | 1 | | 1 (0.5%) | . | | . |
| Leukopenia | . | . | . | . | 1 | | 1 (0.5%) | . | | . |
| Injury, poisoning and procedural complications | . | . | 136 | 55 (27.4%) | . | | . | . | | . |
| Allergic transfusion reaction (anaphylactic reaction, skin reaction) | . | . | 110 | 47 (23.4%) | . | | . | . | | . |
| Febrile nonhemolytic transfusion reaction | . | . | 23 | 14 (7.0%) | . | | . | . | | . |
| Transfusion-related circulatory overload | . | . | 2 | 2 (1.0%) | . | | . | . | | . |
| Delayed haemolytic transfusion reaction | . | . | 1 | 1 (0.5%) | . | | . | . | | . |
| Cardiac disorders/Vascular disorders | 104 | 55 (27.4%) | 14 | 4 (2.0%) | 2 | | 2 (1.0%) | . | | . |
| Arrhythmia | 43 | 29 (14.4%) | . | . | 2 | | 2 (1.0%) | . | | . |
| Cardiac failure | 18 | 18 (9.0%) | . | . | . | | . | . | | . |
| Embolism | 9 | 6 (3.0%) | . | . | . | | . | . | | . |
| Tachycardia | 1 | 1 (0.5%) | 8 | 3 (1.5%) | . | | . | . | | . |
| Mitral valve incompetence | 5 | 5 (2.5%) | . | . | . | | . | . | | . |
| Hypertension | 2 | 2 (1.0%) | 2 | 2 (1.0%) | . | | . | . | | . |
| Left ventricular dysfunction | 4 | 4 (2.0%) | . | . | . | | . | . | | . |
| Cardiomyopathy | 3 | 3 (1.5%) | . | . | . | | . | . | | . |
| Dilatation atrial | 3 | 3 (1.5%) | . | . | . | | . | . | | . |
| Left ventricular dilatation | 3 | 3 (1.5%) | . | . | . | | . | . | | . |
| Flushing | . | . | 3 | 1 (0.5%) | . | | . | . | | . |
| Pericarditis | 2 | 2 (1.0%) | . | . | . | | . | . | | . |
| Aortic valve incompetence | 1 | 1 (0.5%) | . | . | . | | . | . | | . |
| Aortic valve stenosis | 1 | 1 (0.5%) | . | . | . | | . | . | | . |
| Atrial fibrillation | 1 | 1 (0.5%) | . | . | . | | . | . | | . |
| Cardiac ventricular disorder | 1 | 1 (0.5%) | . | . | . | | . | . | | . |
| Cardiomegaly | 1 | 1 (0.5%) | . | . | . | | . | . | | . |
| Diastolic dysfunction | 1 | 1 (0.5%) | . | . | . | | . | . | | . |
| Mitral valve calcification | 1 | 1 (0.5%) | . | . | . | | . | . | | . |
| Palpitations | . | . | 1 | 1 (0.5%) | . | | . | . | | . |
| Peripheral arterial occlusive disease | 1 | 1 (0.5%) | . | . | . | | . | . | | . |
| Sinus tachycardia | 1 | 1 (0.5%) | . | . | . | | . | . | | . |
| Supraventricular extrasystoles | 1 | 1 (0.5%) | . | . | . | | . | . | | . |
| Supraventricular tachycardia | 1 | 1 (0.5%) | . | . | . | | . | . | | . |
| Hepatobiliary disorders | 82 | 62 (30.8%) | . | . | . | | . | . | | . |
| Cholelithiasis | 36 | 35 (17.4%) | . | . | . | | . | . | | . |
| Hepatomegaly | 20 | 20 (10.0%) | . | . | . | | . | . | | . |
| Hepatic fibrosis/Hepatic cirrhosis | 13 | 13 (6.5%) | . | . | . | | . | . | | . |
| Hepatic steatosis | 5 | 5 (2.5%) | . | . | . | | . | . | | . |
| Portal vein thrombosis | 2 | 2 (1.0%) | . | . | . | | . | . | | . |
| Biliary dilatation | 1 | 1 (0.5%) | . | . | . | | . | . | | . |
| Cholangitis | 1 | 1 (0.5%) | . | . | . | | . | . | | . |
| Gallbladder polyp | 1 | 1 (0.5%) | . | . | . | | . | . | | . |
| Haemangioma of liver | 1 | 1 (0.5%) | . | . | . | | . | . | | . |
| Hepatic cyst | 1 | 1 (0.5%) | . | . | . | | . | . | | . |
| Portosplenomesenteric venous thrombosis | 1 | 1 (0.5%) | . | . | . | | . | . | | . |
| Infections and infestations | 10 | 10 (5.0%) | 56 | 51 (25.4%) | 6 | | 5 (2.5%) | . | | . |
| Hepatitis C | . | . | 42 | 42 (20.9%) | . | | . | . | | . |
| Hepatitis B | . | . | 12 | 12 (6.0%) | . | | . | . | | . |
| Hepatitis A | 5 | 5 (2.5%) | . | . | . | | . | . | | . |
| Yersinia infection | . | . | . | . | 4 | | 3 (1.5%) | . | | . |
| Pneumonia | 3 | 3 (1.5%) | . | . | . | | . | . | | . |
| Abdominal abscess | . | . | . | . | 1 | | 1 (0.5%) | . | | . |
| Bacterial infection | . | . | 1 | 1 (0.5%) | . | | . | . | | . |
| Respiratory tract infection | 1 | 1 (0.5%) | . | . | . | | . | . | | . |
| Sepsis | . | . | . | . | 1 | | 1 (0.5%) | . | | . |
| Upper respiratory tract infection | 1 | 1 (0.5%) | . | . | . | | . | . | | . |
| Viral infection | . | . | 1 | 1 (0.5%) | . | | . | . | | . |
| Renal and urinary disorders | 47 | 42 (20.9%) | . | . | 17 | | 17 (8.5%) | 6 | | 3 (1.5%) |
| Nephrolithiasis | 20 | 18 (9.0%) | . | . | 6 | | 6 (3.0%) | 4 | | 3 (1.5%) |
| Hypercalciuria | 14 | 14 (7.0%) | . | . | 3 | | 3 (1.5%) | . | | . |
| Albuminuria | 3 | 3 (1.5%) | . | . | 4 | | 4 (2.0%) | . | | . |
| Renal impairment | 3 | 3 (1.5%) | . | . | 3 | | 3 (1.5%) | . | | . |
| Renal cyst | 4 | 4 (2.0%) | . | . | . | | . | . | | . |
| Hyperuricosuria | 2 | 2 (1.0%) | . | . | . | | . | . | | . |
| Hydronephrosis | . | . | . | . | . | | . | 1 | | 1 (0.5%) |
| Nephrocalcinosis | 1 | 1 (0.5%) | . | . | . | | . | . | | . |
| Proteinuria | . | . | . | . | 1 | | 1 (0.5%) | . | | . |
| Renal colic | . | . | . | . | . | | . | 1 | | 1 (0.5%) |
| Respiratory, thoracic and mediastinal disorders | 25 | 25 (12.4%) | 3 | 3 (1.5%) | . | | . | . | | . |
| Pulmonary hypertension | 24 | 24 (11.9%) | 3 | 3 (1.5%) | . | | . | . | | . |
| Pulmonary oedema | 1 | 1 (0.5%) | . | . | . | | . | . | | . |
| Gastrointestinal disorders | 13 | 10 (5.0%) | 2 | 2 (1.0%) | 7 | | 7 (3.5%) | . | | . |
| Gastritis/Gastric ulcer | 6 | 6 (3.0%) | . | . | 1 | | 1 (0.5%) | . | | . |
| Diarrhoea | 1 | 1 (0.5%) | . | . | 3 | | 3 (1.5%) | . | | . |
| Nausea | . | . | 2 | 2 (1.0%) | . | | . | . | | . |
| Pancreatic atrophy | 2 | 2 (1.0%) | . | . | . | | . | . | | . |
| Abdominal pain upper | . | . | . | . | 1 | | 1 (0.5%) | . | | . |
| Constipation | . | . | . | . | 1 | | 1 (0.5%) | . | | . |
| Duodenitis | 1 | 1 (0.5%) | . | . | . | | . | . | | . |
| Enteritis | 1 | 1 (0.5%) | . | . | . | | . | . | | . |
| Oesophageal varices haemorrhage | 1 | 1 (0.5%) | . | . | . | | . | . | | . |
| Oesophagitis | 1 | 1 (0.5%) | . | . | . | | . | . | | . |
| Vomiting | . | . | . | . | 1 | | 1 (0.5%) | . | | . |
| Ear and labyrinth disorders | 3 | 3 (1.5%) | 1 | 1 (0.5%) | 16 | | 14 (7.0%) | . | | . |
| Deafness neurosensory | 3 | 3 (1.5%) | . | . | 14 | | 14 (7.0%) | . | | . |
| Vertigo | . | . | 1 | 1 (0.5%) | 1 | | 1 (0.5%) | . | | . |
| Tinnitus | . | . | . | . | 1 | | 1 (0.5%) | . | | . |
| Eye disorders | 11 | 9 (4.5%) | . | . | 7 | | 6 (3.0%) | . | | . |
| Cataract | 2 | 2 (1.0%) | . | . | 4 | | 4 (2.0%) | . | | . |
| Chorioretinal atrophy | 6 | 6 (3.0%) | . | . | . | | . | . | | . |
| Diabetic retinopathy | 2 | 2 (1.0%) | . | . | . | | . | . | | . |
| Maculopathy | . | . | . | . | 1 | | 1 (0.5%) | . | | . |
| Retinal degeneration | . | . | . | . | 1 | | 1 (0.5%) | . | | . |
| Retinal pigment epitheliopathy | . | . | . | . | 1 | | 1 (0.5%) | . | | . |
| Strabismus | 1 | 1 (0.5%) | . | . | . | | . | . | | . |
| Musculoskeletal and connective tissue disorders | 12 | 10 (5.0%) | 2 | 1 (0.5%) | 4 | | 4 (2.0%) | . | | . |
| Back pain | 1 | 1 (0.5%) | 2 | 1 (0.5%) | . | | . | . | | . |
| Arthralgia | . | . | . | . | 2 | | 2 (1.0%) | . | | . |
| Arthritis | . | . | . | . | 2 | | 2 (1.0%) | . | | . |
| Hip fracture | 2 | 2 (1.0%) | . | . | . | | . | . | | . |
| Lower limb fracture | 2 | 2 (1.0%) | . | . | . | | . | . | | . |
| Cartilage development disorder | 1 | 1 (0.5%) | . | . | . | | . | . | | . |
| Multiple fractures | 1 | 1 (0.5%) | . | . | . | | . | . | | . |
| Osteonecrosis | 1 | 1 (0.5%) | . | . | . | | . | . | | . |
| Pseudoxanthoma elasticum | 1 | 1 (0.5%) | . | . | . | | . | . | | . |
| Skull malformation | 1 | 1 (0.5%) | . | . | . | | . | . | | . |
| Tibia fracture | 1 | 1 (0.5%) | . | . | . | | . | . | | . |
| Ulna fracture | 1 | 1 (0.5%) | . | . | . | | . | . | | . |
| Skin and subcutaneous tissue disorders | 5 | 4 (2.0%) | 11 | 2 (1.0%) | 1 | | 1 (0.5%) | . | | . |
| Hyperhidrosis | . | . | 11 | 2 (1.0%) | . | | . | . | | . |
| Skin ulcer | 5 | 4 (2.0%) | . | . | . | | . | . | | . |
| Drug eruption | . | . | . | . | 1 | | 1 (0.5%) | . | | . |
| Nervous system disorders | 2 | 2 (1.0%) | 9 | 4 (2.0%) | 2 | | 2 (1.0%) | . | | . |
| Dizziness | . | . | 5 | 2 (1.0%) | . | | . | . | | . |
| Headache | . | . | 4 | 4 (2.0%) | . | | . | . | | . |
| Diabetic neuropathy | 1 | 1 (0.5%) | . | . | . | | . | . | | . |
| Paraparesis | 1 | 1 (0.5%) | . | . | . | | . | . | | . |
| Peripheral sensory neuropathy | . | . | . | . | 1 | | 1 (0.5%) | . | | . |
| Somnolence | . | . | . | . | 1 | | 1 (0.5%) | . | | . |
| Psychiatric disorders | 3 | 3 (1.5%) | 5 | 3 (1.5%) | 1 | | 1 (0.5%) | . | | . |
| Dysphoria | . | . | 5 | 3 (1.5%) | . | | . | . | | . |
| Neuropsychiatric disorder under treatment (not a PT term) | 3 | 3 (1.5%) | . | . | 1 | | 1 (0.5%) | . | | . |
| General disorders and administration site conditions | . | . | 4 | 3 (1.5%) | 1 | | 1 (0.5%) | . | | . |
| Discomfort | . | . | 2 | 2 (1.0%) | . | | . | . | | . |
| Feeling hot | . | . | 2 | 1 (0.5%) | . | | . | . | | . |
| Device related thrombosis | . | . | . | . | 1 | | 1 (0.5%) | . | | . |
| Investigations | 1 | 1 (0.5%) | 2 | 2 (1.0%) | . | | . | . | | . |
| Cold agglutinins positive | . | . | 1 | 1 (0.5%) | . | | . | . | | . |
| Glucose tolerance test abnormal | 1 | 1 (0.5%) | . | . | . | | . | . | | . |
| HTLV test positive | . | . | 1 | 1 (0.5%) | . | | . | . | | . |
| Neoplasms benign, malignant and unspecified (incl cysts and polyps) | 3 | 3 (1.5%) | . | . | . | | . | . | | . |
| Hepatocellular carcinoma | 3 | 3 (1.5%) | . | . | . | | . | . | | . |
| Pregnancy, puerperium and perinatal conditions | 3 | 3 (1.5%) | . | . | . | | . | . | | . |
| Foetal growth restriction | 2 | 2 (1.0%) | . | . | . | | . | . | | . |
| Premature baby | 1 | 1 (0.5%) | . | . | . | | . | . | | . |
| Immune system disorders | . | . | . | . | 2 | | 2 (1.0%) | . | | . |
| Dermatitis allergic | . | . | . | . | 1 | | 1 (0.5%) | . | | . |
| Hypersensitivity | . | . | . | . | 1 | | 1 (0.5%) | . | | . |

HTLV, human T-cell lymphotropic virus; ICT, iron chelation therapy; LLT, Lowest Level Term; MedDRA, Medical Dictionary for Regulatory Activities; n_ev_, number of events; PT, preferred term; SOC, System Organ Class.

**Table SVI.** Association of patient and disease characteristics with the most prevalent disease and/or treatment-related complications

| Parameter | Endocrine/Metabolism and nutrition disorders | | Cardiac/Vascular disorders | | Blood and lymphatic system disorders | | Hepatobiliary disorders | | | Renal and urinary disorders | | |  |
| --- | --- | --- | --- | --- | --- | --- | --- | --- | --- | --- | --- | --- | --- |
|  | OR (95% CI) | *P*-value | OR (95% CI) | *P*-value | OR (95% CI) | *P*-value | | OR (95% CI) | *P*-value | | OR (95% CI) | *P*-value | |
| Male gender (*n* = 201) | 0.92 (0.38–2.22) | 0.855 | 1.56 (0.75–3.24) | 0.236 | 0.73 (0.36–1.48) | 0.387 | | 1.91 (0.91–3.98) | 0.086 | | 1.43 (0.68–3.01) | 0.341 | |
| Age at enrolment >40 years (*n* = 201) | 1.04 (0.39–2.78) | 0.939 | 3.00 (1.00–8.96) | **0.049** | 1.43 (0.63–3.22) | 0.388 | | 0.65 (0.29–1.44) | 0.287 | | 1.11 (0.47–2.63) | 0.818 | |
| Age at enrolment >50 years (*n* = 201) | 2.02 (0.66–6.21) | 0.218 | 1.66 (0.77–3.56) | 0.197 | 1.59 (0.78–3.22) | 0.201 | | 0.39 (0.14–1.05) | 0.063 | | 1.27 (0.58–2.81) | 0.553 | |
| Age at first RBC transfusion >1 year (*n* = 198) | 0.57 (0.24–1.38) | 0.214 | 1.69 (0.81–3.54) | 0.162 | 4.74 (2.19–10.26) | **<0.001** | | 0.98 (0.47–2.04) | 0.948 | | 0.60 (0.28–1.25) | 0.173 | |
| Age at iron chelation therapy onset >5 years (*n* = 192) | 0.72 (0.29–1.77) | 0.471 | 0.95 (0.45–2.01) | 0.888 | 2.00 (0.95–4.19) | 0.067 | | 0.53 (0.25–1.12) | 0.095 | | 0.61 (0.29–1.29) | 0.197 | |
| >40 years elapsed from first RBC transfusion to enrolment (*n* = 198) | 0.92 (0.39–2.19) | 0.852 | 1.26 (0.60–2.63) | 0.544 | 0.33 (0.16–0.65) | **0.002** | | 0.97 (0.46–2.04) | 0.930 | | 1.59 (0.74–3.42) | 0.231 | |
| Time from first RBC transfusion to enrolment (years) (*n* = 198) | 1.01 (0.97–1.04) | 0.714 | 0.99 (0.96–1.02) | 0.592 | 0.94 (0.91–0.96) | **<0.001** | | 1.01 (0.97–1.04) | 0.689 | | 1.02 (0.98–1.05) | 0.319 | |
| Exposure to iron chelation >35 years (*n* = 201) | 0.84 (0.36–1.97) | 0.683 | 1.71 (0.82–3.57) | 0.153 | 0.34 (0.17–0.69) | **0.003** | | 0.80 (0.39–1.67) | 0.555 | | 1.40 (0.67–2.92) | 0.371 | |
| Length of exposure to iron chelation (years) (*n* = 201) | 1.00 (0.96–1.04) | 0.863 | 0.98 (0.95–1.01) | 0.286 | 0.93 (0.90–0.95) | **<0.001** | | 1.02 (0.99–1.06) | 0.233 | | 1.02 (0.98–1.05) | 0.340 | |
| Non-disease related surgical/medical history/comorbidity (*n* = 201) | 2.04 (0.86–4.85) | 0.106 | 2.22 (1.01–4.90) | **0.048** | 2.26 (1.11–4.62) | **0.025** | | 0.87 (0.42–1.80) | 0.700 | | 1.15 (0.55–2.41) | 0.714 | |
| Non-disease related comorbidity burden (*n* = 201) | 2.14 (0.70–6.55) | 0.184 | 3.74 (1.77–7.89) | **<0.001** | 3.56 (1.78–7.11) | **<0.001** | | 0.71 (0.30–1.67) | 0.429 | | 1.64 (0.76–3.53) | 0.207 | |
| History of splenectomy (*n* = 201) | 0.34 (0.13–0.89) | **0.028** | 1.98 (0.93–4.21) | 0.078 | 1.06 (0.55–2.05) | 0.863 | | 0.24 (0.11–0.54) | **<0.001** | | 1.05 (0.51–2.19) | 0.891 | |
| >24 transfusions in the 48 weeks prior to enrolment (*n* = 201) | 0.85 (0.36–2.00) | 0.708 | 0.84 (0.40–1.76) | 0.649 | 0.40 (0.19–0.82) | **0.013** | | 1.80 (0.86–3.75) | 0.117 | | 1.36 (0.65–2.83) | 0.409 | |
| Average pre-transfusion haemoglobin levels in the 48 weeks prior to enrolment >9 g/dL (*n* = 201) | 0.83 (0.23–2.98) | 0.775 | 0.64 (0.25–1.63) | 0.347 | 0.17 (0.07–0.39) | **<0.001** | | 1.37 (0.45–4.23) | 0.580 | | 6.90 (0.91–52.48) | 0.062 | |
| Average serum ferritin levels in  the 48 weeks prior to enrolment  >1000 μg/L (*n* = 200) | 1.82 (0.59–5.61) | 0.296 | 1.43 (0.65–3.18) | 0.377 | 1.44 (0.69–2.99) | 0.328 | | 1.21 (0.54–2.73) | 0.647 | | 0.84 (0.35–1.99) | 0.693 | |
| Normal iron overload grade based on most recently available LIC (*n* = 196) | 1.04 (0.42–2.56) | 0.939 | 0.58 (0.28–1.23) | 0.158 | 0.61 (0.31–1.21) | 0.155 | | 1.02 (0.46–2.24) | 0.967 | | 1.26 (0.57–2.82) | 0.571 | |
| β^+^/β^0^, β^0^/β^0^ vs β^+^/β^+^, other genotype  (*n* = 201) | 1.22 (0.52–2.88) | 0.645 | 0.99 (0.48–2.03) | 0.974 | 0.50 (0.25–0.98) | **0.042** | | 0.92 (0.44–1.91) | 0.827 | | 1.22 (0.59–2.53) | 0.599 | |

CI, confidence interval; LIC, liver iron concentration; OR, odds ratio; RBC, red blood cell. Values in bold indicate statistical significance, i.e., *P* < 0.05.
